# Cardiac regeneration in *Xenopus tropicalis* and *Xenopus laevis*: discrepancies and problems

**DOI:** 10.1186/s13578-018-0230-6

**Published:** 2018-04-23

**Authors:** Souqi Liao, Wenyan Dong, Hui Zhao, Ruijin Huang, Xufeng Qi, Dongqing Cai

**Affiliations:** 1 Key Laboratory of Regenerative Medicine, Ministry of Education, Jinan University, Guangzhou, 510632 People’s Republic of China; 2 Joint Laboratory for Regenerative Medicine, Chinese University of Hong Kong-Jinan University, Guangzhou, 510632 China; 3 International Base of Collaboration for Science and Technology (JNU), Ministry of Science and Technology, Guangzhou, 510632 Guangdong Province China; 4 Department of Developmental and Regenerative Biology, Jinan University, Guangzhou, 510632 China; 5 Stem Cell and Regeneration TRP, School of Biomedical Sciences, Chinese University of Hong Kong, Hong Kong, China; 6 Institute of Anatomy, University of Bonn, Bonn, Germany

**Keywords:** *X. tropicalis*, *Xenopus laevis*, Cardiac regeneration, Heart injury

## Abstract

Two studies have recently focused on adult heart regeneration in *Xenopus*. While we reported on cardiac myogenic regeneration in *Xenopus tropicalis* after injury, Marshall and colleagues found no regeneration in an injured heart in *Xenopus laevis.* Here, we would like to join the discussion initiated by Marshall et al. who debated the methods and species differences in both studies. We agree with their view that the species difference in cardiac regenerative capacity could lead to different results in both of these studies. Moreover, we suggest that the age of the animals used in these studies could lead to differences in regeneration. A 5-year old *X. laevis* is much more advanced in age than a 1-year old *X. tropicalis*. The other reason for the discrepancies could be the size of the clot. Due to different resection protocols, the clot formed after the endoscopic resection performed by Marshall et al. was much larger than that after a conventional resection, as used in our study. Furthermore, the difference in the site of injury could influence the healing and regeneration differences. The influence of the organismal age, techniques used to induce injury and site of injury on regeneration need to be examined in detail to assess the regenerative potential of the amphibian heart.

Dear Editor,

First, thank you for the invitation to provide a response to the Letter-to-the-Editor by Marshall and colleagues: “Is adult cardiac regeneration absent in *Xenopus laevis* yet present in *Xenopus tropicalis*?”. In the letter, the authors compared the methodical approaches carried out in our publication entitled “Heart regeneration in adult *X. tropicalis* after apical resection” [1] and in their publication entitled “Persistent fibrosis, hypertrophy and sarcomere disorganisation after endoscopy-guided heart resection in adult *Xenopus*” [2] and discussed the discrepancy between these studies. Here, we would like to respond to the possible discrepancies regarding *Xenopus* cardiac regeneration described in the two publications.

Some of the aspects discussed in this manuscript are interesting. First, we agree with the view of Marshall and colleagues that the differences in the cardiac regenerative capacity of *X. tropicalis* and *X. laevis* could lead to different results in both of these studies. Although *X. tropicalis* and *X. laevis* belong to the same genus, their genomes are significantly different; *X. laevis* is an allotetraploid, and *X. tropicalis* has a true diploid genome.

After reviewing both articles, we believe that the age of the animals used in these studies could have caused the discrepancies. In Marshall’s study, the author found that no regeneration occurred after cardiac injury in 5-year old *X. laevis* [2]. By contrast, in *X. tropicalis*, we found scar-free regeneration in over 60% of animals subjected to apex resection. The *X. tropicalis* frogs used in our study were all less than 1-year old [1]. The sexual maturity of *X. tropicalis* is approximately 4–6 months, while that of *X. laevis* is approximately 12–18 months. Taking the sexual maturity and average lifespan as standards to evaluate the age difference, the *X. tropicalis* frogs that were used in our study were in the young adult stage, while the *X. laevis* frogs used in the Marshall study were approximately middle-aged or may have been in upper middle age. The regeneration potential is well known to decline with age and increases the fibrosis potential in most tissues and organs. Indeed, in *X. laevis* tadpoles, the tail regeneration ability decreases with age [3]. Hence, the age difference may be a contributing factor that leads to the different outcomes from these two reports.

The operation procedure after heart resection in the two studies may also have caused the different outcomes. We immediately pressed the wound area to stop bleeding, which was not described by Marshall’s group. This difference may result in a significant outcome of a far greater volume of clot formation and far greater volume of bleeding after cardiac damage in the protocol used by Marshall’s group after injury, and these findings are shown in Fig. 1E as well as in the S1-file movie [2]. In terms of the healing and regeneration capacities, an inappropriate volume of clot that fails to dissolve within a suitable time window is well known to be one of the critical factors that causes long-term chronic inflammation and adhesion of wounds, thereby eventually affecting healing and regeneration. Indeed, as shown in our study, we found that two-thirds of the heart after apex resection can undergo full regeneration. Approximately one-third of the amputated heart is only able to recover in a nearly heart-shaped morphology but contains adhesions between the regenerated site and peripheral tissues. We speculate that this finding is due to inappropriate adhesion with peripheral tissues. It is possible that incomplete haemostasis and the incurring chronic inflammation environment cause the remaining scar after heart regeneration. However, the mechanism underlying this process remains elusive. In the study conducted by Marshall’s group, a similar phenomenon was observed as well.

The location of the injury site could also impact regeneration. The location of the amputation in our protocol was at the apex [Approximately 10% (approximately 1 mm in length) of ventricle tissue from the cardiac apex]. According to the protocol used by Marshall’s group, the amputation was carried out in the apex; however, we found that the injury was mainly located in the middle part of the anterior wall of the ventricle (Figure 2) [2]. The difference in the injured location may cause differences in healing and regenerated. We, as well as others, have reported that in a rodent model, the distribution and density of endogenous cardiac stem cells and cardiac telocytes, which are important for myocardial regeneration, are different in the atrium-atria region, medium region and base (including the apex area). The density of both types of cells in the medium region of the heart (mainly in the anterior and posterior wall of the ventricle) is lower than that of the atrium-atria part and base [4, 5]. Hence, the difference in the cardiac regeneration potential between our study in *X. tropicalis* and Marshall’s study group in *X. laevis* may also be due to the different location of the injury in the myocardium.

While the different outcomes in the two studies are potentially interesting and difficult to comprehend at the moment, we disagree with Marshall’s concern regarding our observation of the cardiac regeneration potential in *X. tropicalis*. We believe that we observed cardiomyocyte proliferation during regeneration. We used phospho-histone H3, anti-alpha skeletal muscle actin (α-SA) and DAPI triple staining to semi quantify the proliferation of endogenous cardiomyocytes in the regenerated area and near the wound area in situ. Only nuclei that were positive in the three staining assays and were embedded with α-SA positive cytoplasm were counted. Since the sections in our study had a thickness of 5 µm, the probability that the nuclei of cardiomyocytes were identified as nuclei of other cells was very low. Therefore, as α-SA is marker of non-mature and mature cardiomyocytes, it should effectively exclude most of the other non-cardiomyocytes (such as fibroblasts and inflammatory cells). We found that the density of PH3^+^/α-SA^+^-positive cells was significantly increased in the regenerated area and near the wound area between 4 and 16 days after apical resection. The peak of PH3^+^/α-SA^+^-positive cells was observed between 8 and 16 days after apical resection. At 4 and 8 days after apical resection, some PH3^+^/α-SA^+^-positive cardiomyocytes with a disorganized cytoskeletal morphology were found within the regenerated area, and many PH3^+^/α-SA^+^-positive spindle cells were found in the regenerated area located within the area of the epicardial tissue. At 30–60 days after apical resection, the density of PH3^+^/α-SA^+^-positive cells in the regenerated myocardium was significantly decreased. Between 30 and 60 days, the amputated area was regenerated by new cardiomyocytes with mature cardiac phenotypes, as indicated by their regular cardiac-specific cross-striated morphology and cardiac troponin T staining. All of these observations suggest an important role of the proliferation of endogenous cardiomyocytes during the regeneration of the injured adult *X. tropicalis* myocardium [1].

According to the observations made by Marshall et al., adult *Xenopus* cannot regenerate injured heart muscle. These findings suggest that the wound in the myocardium was mainly repaired through cardiac muscle hypertrophy. The possibility of heart muscle regeneration through cardiomyocyte proliferation was excluded since sarcomere disorganization was observed in the boundary region between the normal myocardium and wound in adult *X. laevis*. Furthermore, the coupling of dedifferentiation and proliferation is a conserved mechanism for muscle regeneration [6]. However, in situ proliferation of cardiomyocytes was not observed. The entire ventricle was collected to conduct q-PCR analysis of the proliferation of the myocardium while, according to the protocol, only approximately 4% of injury occurred in the myocardium. Therefore, q-PCR for entire ventricle is not a reliable approach to reflect possible heart regeneration, which only occurs at the injury region. Indeed, according to our in situ observation using PH3 + α-SA + DAPI- and PH3 + cTnT + DAPI-immunostaining, proliferating cells are only found in the boundary of the wound region. The very small proportion of proliferating cardiomyocytes in the entire heart muscle may be the reason why the difference in gene expression for cell proliferation in the entire ventricle was not detected in Marshall’s study.

Our observations were recently independently confirmed by another group at our institute (unpublished data). This group carried out the experiment using the same resection protocol published in our paper. They obtained similar findings by using 6-month-old *X. tropicalis*, e.g., approximately two-thirds of amputated hearts regenerated with a perfect heart-shaped morphology, while approximately one-third of amputated hearts recovered a nearly heart-shaped morphology and contained adhesions between the regenerated site and peripheral tissues. Of note, in addition to pH3^+^/α-actinin^+^/DAPI^+^ staining, proliferating cardiomyocytes were identified by EDU^+^/α-actinin^+^/DAPI^+^ and Mef2c^+^/PCNA^+^/DAPI^+^ staining. Using these immunostaining methods, numerous proliferating cardiomyocytes were identified in the wound of the injured myocardium (personal communication).

In summary, we agree that there are discrepancies between the two studies and that further detailed studies are needed to clarify these differences. We believe that the differences in the age of the animals used, techniques used to induce the formation of clots and location that was injured could be some of the reasons for the different regenerative outcomes after adult myocardial injury in our study and in Marshall et al.’s report. We hope that these discussions will encourage new investigations to delineate the influence of age, location of injury and adhesions and species on the regeneration of the *Xenopus* heart. These findings may lead to the discovery of potentially novel mechanisms for heart regeneration.

## Abbreviations

*X. tropicalis*: *Xenopus tropicalis*
*X. laevis*: *Xenopus laevis*
PH3: phospho-histone H3
α-SA: alpha skeletal muscle actin
DAPI: 4,6-diamidino-2-phenylindole
EDU: 5-ethynyl-2′-deoxyuridine
Mef2c: myocyte-specific enhancer factor 2C
PCNA: proliferating cell nuclear antigen
